# Benefits of Steroid Therapy in COVID-19 Patients with Different PaO_2_/FiO_2_ Ratio at Admission

**DOI:** 10.3390/jcm10153236

**Published:** 2021-07-22

**Authors:** Serena Vita, Daniele Centanni, Simone Lanini, Pierluca Piselli, Silvia Rosati, Maria Letizia Giancola, Annalisa Mondi, Carmela Pinnetti, Simone Topino, Pierangelo Chinello, Silvia Mosti, Gina Gualano, Francesca Faraglia, Fabio Iacomi, Luisa Marchioni, Micaela Maritti, Enrico Girardi, Giuseppe Ippolito, Emanuele Nicastri, on behalf of the ReCOVeRI Study Group

**Affiliations:** National Institute for Infectious Diseases, Lazzaro Spallanzani, IRCCS, Via Portuense, 292, 00149 Rome, Italy; serena.vita@inmi.it (S.V.); Simone.lanini@inmi.it (S.L.); pierluca.piselli@inmi.it (P.P.); silvia.rosati@inmi.it (S.R.); mletizia.giancola@inmi.it (M.L.G.); annalisa.mondi@inmi.it (A.M.); carmela.pinnetti@inmi.it (C.P.); simone.topino@inmi.it (S.T.); pierangelo.chinello@inmi.it (P.C.); silvia.mosti@inmi.it (S.M.); gina.gualano@inmi.it (G.G.); francesca.faraglia@inmi.it (F.F.); Fabio.iacomi@inmi.it (F.I.); luisa.marchioni@inmi.it (L.M.); micaela.maritti@inmi.it (M.M.); enrico.girardi@inmi.it (E.G.); giuseppe.ippolito@inmi.it (G.I.); emanuele.nicastri@inmi.it (E.N.)

**Keywords:** steroids, COVID-19, Sars-CoV-2 pneumonia, ARDS

## Abstract

Introduction: The use of steroid therapy in patients within the context of SARS-CoV-2 infection is still a matter of debate. This study aimed to evaluate if potential steroid benefits could be predicted by the ratio of arterial oxygen partial pressure (PaO_2_ in mmHg) to fractional inspired oxygen (FiO_2_) (P/F) in COVID-19 patients at admission. Materials and Methods: Medical records were retrospectively collected from all adult patients admitted because of COVID-19 from 29 January to 31 July 2020. The association of steroid therapy with 28-day all-cause mortality outcome was analysed in a multivariable logistic regression model adjusted for confounding factors. Results: Overall, 511 patients were analysed, of which 39.1% underwent steroid therapy. Steroid treated patients were mostly male, older, and more frequently treated with antiviral drugs and aminoquinolines; the most common comorbidities were hypertension, followed by cardiovascular disease. Overall, 51 patients died within 28-days, and overall 28-days mortality was 19.5% in the cohort of patients exposed to steroids versus 3.9% mortality in unexposed patients (*p* < 0.001). Steroid therapy on patients with P/F ratio of 235 mmHg or higher at admission can be considered as detrimental, with an 8% increased probability of death. Conclusions: Steroid therapy is associated with increased 28-day mortality in COVID-19 in patients with mild or no ARDS.

## 1. Introduction

In December 2019 a novel viral agent named Severe Acute Respiratory Syndrome CoronaVirus-2 (SARS-CoV-2) was identified as the etiologic agent of the CoronaVIrus Disease-19 (COVID-19) outbreak occurring in Wuhan, China [1]. 

The clinical spectrum of COVID-19 ranges from asymptomatic to more relevant clinical forms: about 80% of COVID-19 cases are asymptomatic, paucisymptomatic, or exhibits mild to moderate symptoms; 15% progresses to severe pneumonia; and a further 5% develops acute respiratory distress syndrome (ARDS), septic shock, and/or multiple organ failures related to dysregulated systemic inflammation highlighted by increases in the serum levels of inflammatory cytokines [2]. Indeed, although the pathophysiology of COVID-19 remains incompletely understood, lung injury is not only associated with direct viral pathogenicity, but even to hyper immune response leading to a release of a large number of pro- and anti-inflammatory cytokines. This increased production of cytokines results in a destruction of low tract respiratory airway and alveolar and vascular endothelium, proceeding to a local inflammation with pulmonary oedema and hyaline membrane formation [3,4]. 

Corticosteroids, because of their anti-inflammatory, antioxidant, pulmonary vasodilator, and anti-oedematous effects, have been tested in different scenarios of ARDS [5] and could be considered a suitable therapy for these patients. Indeed, they are able to down-regulate the systemic inflammation [6] and their use has been associated (at lung level) with substantial improvement in the indices of alveolar–capillary membrane permeability and the mediators of inflammation and tissue repair [7]. However, results on the benefit of steroid use on the diseases progression/prognosis are still controversial. 

The first randomized clinical trial to report that the use of dexamethasone reduced 28-day mortality in patients requiring oxygen therapy or mechanical ventilation was the RECOVERY study [8]. Later on, a prospective meta-analysis of seven randomized clinical trials showed that the administration of corticosteroids was associated with lower 28-day all-cause mortality in critically ill patients with COVID-19 [9]. In contrast, Liu and colleagues [10] published data from a Chinese multicentre cohort showing that the administration of corticosteroids in severe COVID-19–related ARDS was associated with increased mortality and delayed SARS-CoV-2 coronavirus RNA clearance. A recent meta-analysis with a sample size of 5270 patients suggested that treatment with steroids was associated with increased prolonged hospital stays and mortality in patients with SARS-CoV-2 pneumonia [11]. The same results on mortality were reported by Bartoletti et al. in Italy [12].

The aim of this study was to evaluate if, in COVID-19 patients, the potential steroid benefits could be predicted by the ratio of arterial oxygen partial pressure (PaO2 in mmHg) to fractional inspired oxygen (FiO_2_) (P/F ratio) at admission. The starting assumption is that 28-day all-cause mortality is directly associated with steroid therapy and P/F, and the effect of the latter is heterogeneous between patients who did and did not undergo steroid therapy.

## 2. Materials and Methods

### 2.1. Study Design and Setting 

This was a single center cohort study conducted at the National Institute for Infectious Diseases “Lazzaro Spallanzani” in Rome, Italy. All consecutive adult patients admitted because of COVID-19 from 29 January to 31 July 2020 were included. The present study was conducted to determine if the effect of corticosteroid therapy on 28-day mortality among patients who received steroids and those who did not could be predicted by P/F ratio at admission.

### 2.2. Participants 

The medical electronical records of clinical, demographic, and laboratory data were retrospectively collected from all adult patients. For patients able to provide a signed informed consent (IC) at the time of hospital admission, written IC was obtained prior to data collection. Otherwise, patients consented as soon as they were able to sign. The referral Ethics Committee approved the study. All research was conducted in accordance with the Declaration of Helsinki. This study is reported in compliance with the STROBE statement [13].

The inclusion criteria were that patients have a Sars-CoV-2 infection (with a RT-PCR positive on nasopharyngeal swabs or lower tract sample) and that they underwent steroid therapy. Moreover, patients must have received an actual measurement of the P/F ratio at the first day of hospitalization or within 2 days of admission.

To count as steroid therapy and be included in the analysis, patients must have been treatment with any steroid drug in the form of intravenous methylprednisolone (1 mg/kg per day), intravenous, liquid or oral dexamethasone (6 mg per day), or oral prednisolone (1 mg/kg per day) within 7 days of admission for at least two full days. It was permitted to switch between the routes of administration and the type of steroid drug according to clinical circumstances during the hospital stay.

Patients who were already on chronically oral or intravenous steroid therapy and patients that died or were discharged within the first 48 h of admission were excluded. The patients were classified into two cohorts, based on their exposure to steroids during hospitalization. Patients were followed until discharge or death.

### 2.3. Variables

Data for each patient was analysed and, for each patient, the following information was extracted from electronic clinical record: age at admission (years, as a categorical variable with three levels); gender (binary); days since COVID-19 symptoms onset; the date of steroid administration; and the ratio of arterial oxygen partial pressure (PaO_2_ in mmHg) to fractional inspired oxygen (FiO_2_) (P/F) (continuous) at the day of admission. 

Other variables (binary) dealing with the health status and drug use of the patients were included: the presence of hypertension, diabetes, chronic obstructive pulmonary disease (COPD), or cardiovascular disease (CVD) (coronary heart disease, heart failure, with/without cerebrovascular disease); obesity; cancer; and renal failure; were defined according to the self-reported medical history of each patient. Drug use variables included the use of: antivirals (lopinavir/ritonavir, darunavir/ritonavir, remdesevir); aminoquinolines (chloroquine and hydroxychloroquine); and anti-IL6 monoclonal antibodies (i.e., tocilizumab).

The primary endpoint was the 28-day all-cause mortality, which was treated as the primary outcome. Steroid therapy and the P/F ratio at admission were considered as the main predictors, leaving all previous variables as potential confounders. In addition, the interaction between steroid therapy and the P/F ratio ad admission was treated as an effect modifier. Intensive care unit (ICU) admission or all-cause mortality within 28 days were studied as a combined outcome in a sensitivity analysis for 28-day all-cause mortality.

The clinical criteria for ICU admission were based on the Surviving Sepsis Campaign guidelines on the management of critically ill adults with COVID-19 published on 28 March 2020 [14].

### 2.4. Bias

As in every cohort study, such a study could suffer from bias. Due to the method used for identifying confounding factors, confounding bias could arise. As a matter of fact, the identification of confounding factors has been based upon the compatibility of the data with the null hypothesis (no association between the outcome and the confounding factor). Such compatibility was measured by *p*-value, which suffers from its limitations, as well as the sample size. In addition, a selection bias could arise since we excluded patients with no actual measurement of P/F ratio at admission.

### 2.5. Statistical Methods

All data analyses were carried out by STATA 15 statistical package (Stata Corp LP, College Station, TX, USA). The association of steroid therapy on 28-day all-cause mortality outcome was analysed in a multivariable logistic regression model together with P/F ratio at admission, and adjusted for confounding factors. The set of potential confounders was selected through separate bivariate logistic regression models involving 28-day all-cause mortality and steroid therapy as outcomes. Confounders whose association with outcomes was found to be statistically significant (*p*-value < 0.05) for both bivariate models were added to the multivariable logistic model. Eventually, confounders which showed no significant association (*p*-value > 0.05) in the adjusted model were removed (Figure S1).

In order to assess the completeness of the adjusted model just found, likelihood ratio tests were performed between the model and the one including other confounders not selected before. No likelihood ratio test was proven to be significant (data not shown).

In order to improve the prediction of the model, a sensitivity analysis was conducted involving the composite outcome made by death or ICU admission within 28-days. The confounding variable selection was performed similarly to the first analysis, with death within 28 days as the outcome. Likelihood ratio tests were carried out between unadjusted and adjusted models involving one confounder at time. Only confounding variables in significant tests were promoted to the adjusted model.

Model-based punctual estimates, for exposed and unexposed, as an average of all patients’ covariates and their differences, were obtained by marginal predictions with the calculation of 95% CI and corresponding *p*-values.

## 3. Results

### 3.1. Study Population

**Figure 1:**
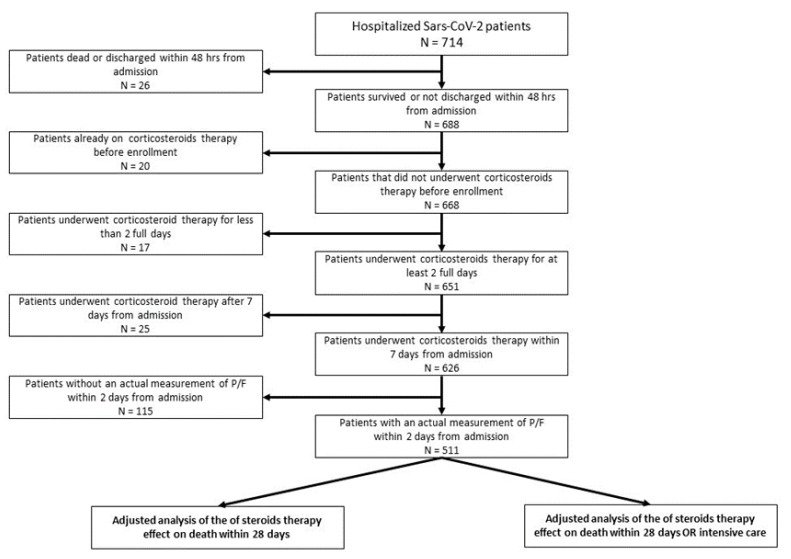
Flow chart of the present study.

A total of 714 consecutive patients, who were admitted at INMI from 29 January to 31 July with virologically confirmed SARS-CoV-2 positive molecular assay on nasopharyngeal swab or by a serological assay, were initially considered. We excluded 26 patients for a short hospital stay (<2 days), 20 patients for the chronic use of steroids, 17 patients for a short use of steroids (less than 2 days), 25 patients for a use of steroids after 7 or more days since admission, and 115 patients with no measurement of the PaO_2_/FiO_2_ ratio within 2 days of admission. A final group of 511 patients with Sars-CoV-2 was analyzed (Figure 1). Among them, 471 patients (92.2%) had pneumonia. The median and IQR of follow-up duration was 14 days (9–21).

### 3.2. Use of Steroids

Among 511 patients, 200 (39.1%) patients underwent therapy during the admission. Of them, 198 (99%) had pneumonia.

As can be seen in Table 1 patients on steroids (“CS Yes”) were mostly male (135 patients, 67.5%), and elderly (98 patients, 49% with an age > 70 years old). The most common disease among treated patients was hypertension (104 patients, 52%), followed by CVD (67 patients, 33.5%). Steroid-treated patients were more frequently treated with antiviral drugs (160 patients, 80%) and with aminoquinolines (120 patients, 60%). Finally, lower P/F ratios at admission were reported in steroid-treated patients than in untreated patients (median (IQR) 283 (199–344) vs. 376 (319–438) mmHg, respectively). The median time from illness onset to steroid therapy was 10 (7–14 IQR) days. The median duration of steroid therapy was 11 (7–15 IQR) days. Overall, 186 cases (93%) received intravenous methylprednisolone. The length of the hospital stay was 18 days (13–30 IQR) for steroid treated patients compared to 11 days (7–16 IQR) for untreated patients (data not shown). Further information on the demographic and clinical features of the study patients are listed in Table 1. In addition, a clearer view of the steroid cohort with respect to the ARDS categories is provided in Table 2 and in a box plot in Supplementary Materials as Figure S2. 

### 3.3. Mortality at Day 28

In the steroid cohort, 39 patients (19.5%) died at a median (IQR) of 14 days (8–23) since admission; whereas, in the untreated cohort, 12 patients (3.9%) died at a median (IQR) of 9.5 days (6–13.5). Thirty-nine patients (76.5%) who have died were >70 years old. Regarding comorbidities, 35 patients (68.6%) had a history of hypertension, 33 patients (64.7%) had a history of CVD, and 14 patients (27.5%) had a history of obesity (Table 2). Finally, non-survivors had a lower P/F at admission than those reported in survivors: median (IQR) 221 (144–329) vs. 352 (188–410) mmHg.

Bivariate analysis with steroid therapy and death within 28 days as separated outcomes showed different associations with observed confounding factors. Confounding factors associated with steroid therapy and death were: age categories (*p*-value < 0.001 for both); COPD (*p*-values: 0.016 and 0.009 for steroids and death, respectively); hypertension (*p*-value < 0.001 for both); CVD (*p*-values: 0.027 and < 0.001 for steroids and death, respectively); and obesity, with a less significant association with steroid therapy than death (*p*-values: 0.060 and 0.002, respectively).

Several likelihood-ratio tests were performed in order to select the right confounding variable to be added to the adjusted model as mentioned in the statistical methods section. The confounding variables whose likelihood-ratio tests were proven to be significant were: the use of monoclonal antibodies (*p*-value = 0.018), age categories (*p*-value < 0.001), diabetes (*p*-value = 0.012), cancer (*p*-value = 0.05), CVD (*p*-value < 0.001), and obesity (*p*-value = 0.013). Nevertheless, only CVD, obesity, and age categories were added to the adjusted final model, but in order to assess the completeness of the adjusted model, several likelihood-ratio tests were performed comparing the adjusted model and the adjusted model with confounding factors not previously selected. The evidence shows that there were no more confounding variables to be added to the adjusted model (data not shown).

Eventually, average marginal predictions were calculated in order to return the heterogeneous effect of steroid therapy on the probability of the outcome, i.e., death within 28 days, with respect to P/F at admission. As shown in Table 3, it starts from a non-significant (*p*-value = 0.875) difference of −2.8% (−37.8%; +32.2% CI) between treated and untreated patients’ mortality curves at P/F equals to 60 mmHg, developing to a significant (*p*-value = 0.010) difference of 7.3% (1.8%; 12.9% CI) at P/F equals to 385 mmHg. Average estimates shown in Table 3 were obtained by calculating the average of the following expression:

(1) $$\mathrm{Probability}\;\mathrm{of}\;\mathrm{death}\;\mathrm{within}\;\mathrm{day}\;28\;\;=\frac{0.989^{x_{\mathrm{pf}}}\times 0.616^{x_{\mathrm{steroid}}}\times 1.01^{x_{\mathrm{int}}}\times 3.969^{x_{\mathrm{cvd}}}\times 3.605^{x_{\mathrm{obese}}}\times 0.676^{x_{\mathrm{age}1}}\times 2.182^{x_{\mathrm{age}2}}\times 0.495}{1+0.989^{x_{\mathrm{pf}}}\times 0.616^{x_{\mathrm{steroid}}}\times 1.01^{x_{\mathrm{int}}}\times 3.969^{x_{\mathrm{cvd}}}\times 3.605^{x_{\mathrm{obese}}}\times 0.676^{x_{\mathrm{age}1}}\times 2.182^{x_{\mathrm{age}2}}\times 0.495}$$

where the bases of the exponentials represent the odds ratios related to the covariates which are shown as an exponent, $x_{pf}$ is related to the P/F ratio at admission and it can assume values from 60 to 385; $x_{steroid}$ is related to the corticosteroid therapy and it can assume values 1 or 0 (whether the patient did or did not undergo steroid therapy, respectively); $x_{int}$ represents the covariate related to the interaction term between P/F at admission and corticosteroid therapy; $x_{cvd}$ and $x_{obese}$ are the binary covariates related to patients’ comorbidities, i.e., CVD and obesity, respectively; $x_{age1}$ and $x_{age2}$ are the covariates related to the patients’ age, the former is 1 (whether the patient’s age is between 50 years and 70 years), 0 otherwise; the latter, on the other hand, is 1 (whether the patient’s age is 70 or more), 0 otherwise.

**Figure 2:**
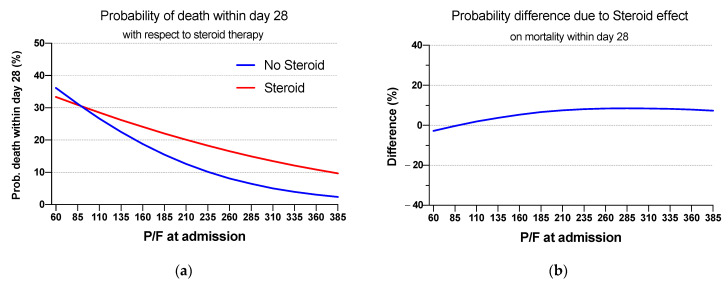
(**a**) Estimated probability of death within 28 days with respect to P/F values and steroid therapy. (**b**) Estimated difference in mortality within 28 days between exposed and unexposed cohort. Light blue shaded area shows confidence interval.

In addition, it can be seen from Table 3 that estimates related to a P/F of 235 mmHg or higher were found to be statistically significant, and from Figure 2a,b it can be deduced that the effect of steroid therapy on patients with a P/F ratio at admission of 235 mmHg or higher can be considered detrimental. Odds ratios obtained from such analyses are 3.97 (1.89–8.32, 95% CI) and 3.60 (1.58–8.23, 95% CI) respectively for CVD and obesity; a complete view of the obtained odds ratio is presented in the Supplementary Materials as Table S1 and Figure S3. Furthermore, in Appendix A, a sensitivity analysis involving age as a continuous variable is presented.

### 3.4. Admission to Intensive Care Unit or Mortality at day 28

Ninety-five patients (18.6%) were admitted to the ICU or died, 52 of them (54.7%) older than 70 years. Regarding comorbidities, 54 (56.8) patients had hypertension, 44 (46.3%) had CVD, and 22 (23.2%) had a history of obesity. Seventy-one (74.7%) patients were treated with antivirals, 52 (54.7%) with aminoquinolines, 43 (45.3%) with azithromycin, and 33 (34.7%) with monoclonal antibodies (Table 4). Finally, patients admitted to the ICU or deceased within 28 days had a median P/F at admission (IQR) of 244 (148–329) mmHg vs. 356 (296–419) mmHg in patients not reaching the combined outcome. 

**Figure 3:**
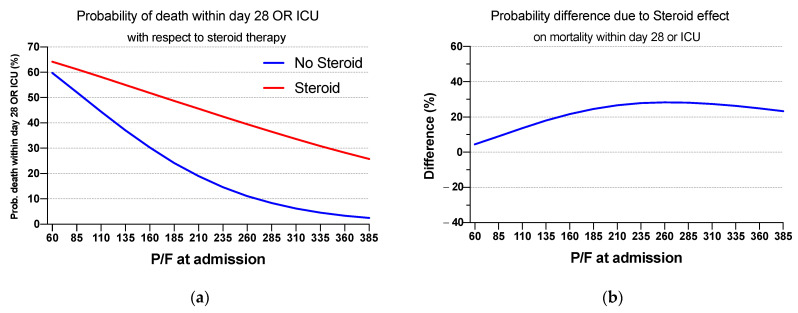
(**a**) Estimated probability of death within 28 days or ICU with respect to P/F values and steroid therapy. (**b**) Estimated difference in mortality within 28 days or ICU admission between exposed and unexposed cohort. Light blue shaded area shows confidence interval.

We performed a sensitivity analysis involving mortality or ICU admission within 28 days as the outcome. The adjusted logistic regression model eventually included CVD, obesity, and age categories as confounders, as seen in the previous adjusted model in Section 3.3. As reported in Table 5 and Figure 3, the difference between treated and untreated patients is wider than the one related to death within 28 days as a single outcome, especially in the right side of the curve concerning high values of P/F, reaching 7.3% (95% CI: 1.8–12.9%) for death within 28 days versus 23.3% (14.3–32.2%) for the composite outcome. In addition, a larger portion of the curve shows a significant difference in the composite outcome probability; as a matter of fact, the curves for treated and untreated patients related to the composite outcome become significant at P/F values of about 170 mmHg or higher as seen in Figure 3b. The average marginal predictions have been calculated in the same way as in the analysis with death within 28 days as a single outcome, by averaging the following expression, including different odds ratios:

(2) $$\mathrm{Probability}\;\mathrm{of}\;\mathrm{death}\;\mathrm{within}\;28\;\mathrm{days}\;\mathrm{OR}\;\mathrm{Intensive}\;\mathrm{Care}\;\mathrm{Unit}\;=\frac{0.987^{x_{\mathrm{pf}}}\times 0.768^{x_{\mathrm{steroid}}}\times 1.01^{x_{\mathrm{int}}}\times 2.746^{x_{\mathrm{cvd}}}\times 2.610^{x_{\mathrm{obese}}}\times 0.760^{x_{\mathrm{age}1}}\times 0.774^{x_{\mathrm{age}2}}\times 2.777}{1+0.987^{x_{\mathrm{pf}}}\times 0.768^{x_{\mathrm{steroid}}}\times 1.01^{x_{\mathrm{int}}}\times 2.746^{x_{\mathrm{cvd}}}\times 2.610^{x_{\mathrm{obese}}}\times 0.760^{x_{\mathrm{age}1}}\times 0.774^{x_{\mathrm{age}2}}\times 2.777}$$

where the same rule as in the probability of death within 28 days was used. Odds ratios obtained from such analyses are 2.75 (1.46–5.18, 95% CI) and 2.61 (1.27–5.34, 95% CI), respectively, for CVD and obesity; a complete view of the obtained Odds ratio is presented in the Supplementary Materials as Table S2 and Figure S4.

## 4. Discussion

Our cohort study suggests that COVID−19 patients with P/F > 235 mmHg at admission treated with steroids had a significantly increased risk of death by day 28 than steroid-untreated patients. Such an unfavorable clinical result is more evident in the sensitivity analysis using death within 28 days or ICU admission as a composite outcome. Indeed, a higher risk of ICU admission or death had been reported in steroid-treated patients with P/F ≳ 170 mmHg at admission. 

COVID−19 disease progression seems to be related to an excessive and uncontrolled immune response driving morbidity and mortality in the most severe cases. Therefore, steroids have been extensively used during the SARS-CoV-2 pandemic. Since March 2020, the Surviving Sepsis Campaign (SSC) guidelines updated the management of critically ill adults with COVID-19 [14]. The experts’ panel supported a weak recommendation to use steroids in patients with COVID-19 and ARDS. The use of corticosteroids in COVID patients with ARDS is recommended at a lower dosing and for a shorter treatment course than in viral pneumonia patients with ARDS. In COVID patients with respiratory failure with no evidence of ARDS, the recommendation is against the routine use of systemic corticosteroids [14]. Indeed, the panel performed a meta-analisys on viral pneumonia (i.e., influenza virus, coronaviruses, and others), showing an association between corticosteroid use and increased mortality. To date, among COVID-19 patients, two randomized trials showed conflicting results [8,15]. The RECOVERY trial found that patients receiving 6 mg of dexamethasone for 10 days had a lower mortality rate compared to controls [8]; the reduction in mortality with dexamethasone was seen only in patients who required supplemental oxygen with or without invasive mechanical ventilation. No benefit of dexamethasone was seen in patients who did not require supplemental oxygen at enrolment. Conversely, the METCOVID trial found no survival benefit of a short course of methylprednisolone (0.5 mg/kg twice daily for 5 days) in patients with COVID-19 pneumonia [15]. Similarly, a multicentre observational study did not find a lower mortality rate in steroid-treated COVID-19 patients [12].

In our study, we did not find a beneficial effect in patients requiring oxygen support with mild ARDS (with P/F between 235 and 300 mmHg), while in patients without ARDS, a detrimental effect was found. Patients with a P/F at admission lower than 235 mmHg experienced a general beneficial, although not statistically significant, effect.

Our data are in line with the SSC guidelines [14]; indeed, the use of steroid in patients without ARDS is not beneficial and is even detrimental, while in patients with ARDS, it seems to have an advantageous effect.

Divergence data from the RECOVERY trial could be related to different causes: First, most of our patients were treated with intravenous methylprednisolone by using a dosage of 1 mg/kg per day higher than the equivalent doses of dexamethasone. Second, in our cohort, the interval between the symptoms’ onset and the steroid administration was 10 days (IQR 7–14) vs. 8 days (IQR 5–13) in the RECOVERY trial. Third, the duration of the steroid therapy was slightly longer in our patients: 11 days (IQR 7–15) vs. 7 days (IQR 3–10), respectively.

Consistent with the data from the RECOVERY trial, a detrimental effect was found in COVID-19 patients without evidence of ARDS and in need of supplemental oxygen therapy. The steroid-driven immune impairment may lead to the prolonged clearance of SARS-CoV-2 RNA from the blood [16], respiratory tract [17], and feces [18]. Considering all these data, the use of steroid therapy in patients with pauci-symptomatic infections of SARS-CoV-2 needs to be strongly discouraged. General practitioners or family doctors involved in the home management of COVID patients need to consider the impact of the COVID-19 social media infodemic in the general population, and support the recommended use of steroids as a life-saving therapy only in COVID-19 inpatients with impaired respiratory functionality.

The results of our analysis should be evaluated in light of the potential limitations due to the study design and the information sampling framework, which is mainly based on real clinical data. The association between the use of steroids and baseline P/F ratio might be biased by the observational design of the study; indeed, patients always underwent medical intervention (including the use of steroids) according to clinical judgment only. Secondly, the study’s external validity might be weakened by the monocentric study design. Thirdly, as for other nonrandomized studies, adjusted analysis may be incomplete, as the result of the imbalanced distribution of unmeasured/unknown confounders (observation bias due to the imbalance of latent variables). Fourthly, no information in terms of the optimal duration of corticosteroid treatment or of the effectiveness of corticosteroid doses according to different P/F ratios is given. Finally, a number of subjects were not included in the study due to the lack of a measure of P/F ratio at admission. Most of these subjects were affected with mild COVID-19 and neither underwent ABG analysis nor steroids therapy and, in addition, data on DNI/DNR orders have not been collected. Furthermore, there could be a potential for bias regarding patients who develop ARDS in the course of the disease (after the initial measurement of PaO_2_/FiO_2_ ratio) and therefore receive steroids. These patients may have a higher mortality risk primarily because of the ARDS, and the effect of the steroids may be overestimated. Despite these limitations, the study provides additional evidence on the use of steroids in COVID-19 patients by using real clinical data. Consistent with other clinical experience, our study claims that the use of steroids should be limited to hospitalized patient with moderate/severe COVID-19 with low a P/F ratio and the need for oxygen supplementation. The study also has some strengths, including a large sample size, a focus on steroid therapy for patients with or without ARDS, and the representativeness of real-life patients.

## 5. Conclusions

In conclusion, steroid therapy may save lives among COVID-19 inpatients with a low P/F ratio at admission, but it could lead to higher mortality among COVID-19 inpatients with mild ARDS or with no need of oxygen therapy.

## Figures and Tables

**Table 1:** Patients’ demographic, co-morbidities, and univariate analysis results with respect to steroid therapy and death within 28 days.

Patients Feature		Total N (%)	CS Yes N (%)	CS No N (%)	OR (95% CI)	*p*-Value	Decd N (%)	Surviv. N (%)	OR (95% CI)	*p*-Value
Age	<50 Years	110 (21.5)	29 (26.4)	81 (73.6)		<0.001	3 (2.7)	107 (97.3)		<0.001
	50–70 Years	198 (38.7)	73 (36.9)	125 (63.1)	1.63 (0.98–2.72)		9 (4.6)	189 (95.5)	1.70 (0.45–6.41)	
	>70 Years	203 (39.7)	98 (48.3)	105 (51.7)	2.61 (1.57–4.32)		39 (19.2)	164 (80.8)	8.48 (2.56–28.14)	
Sex	Female	181 (35.4)	65 (35.9)	116 (64.1)	ref	0.267	19 (10.5)	162 (89.5)	ref	0.774
	Male	330 (64.6)	135 (40.9)	195 (59.1)	1.24 (0.85–1.8)		32 (9.7)	298 (90.3)	0.92 (0.5–1.67)	
use of Antivirals	No	185 (36.2)	40 (21.6)	145 (78.4)	ref	<0.001	15 (8.1)	170 (91.9)	ref	0.281
	Yes	326 (63.8)	160 (49.1)	166 (50.9)	3.49 (2.31–5.27)		36 (11.0)	290 (89.0)	1.41 (0.75–2.65)	
use of Aminoquinoline	No	218 (42.7)	80 (36.7)	138 (63.3)	ref	0.329	22 (10.1)	196 (89.9)	ref	0.942
	Yes	293 (57.3)	120 (41.0)	173 (59.0)	1.20 (0.83–1.72)		29 (9.9)	264 (90.1)	0.98 (0.55–1.76)	
use of Azithromycin	No	327 (64.0)	115 (35.2)	212 (64.8)	ref	0.015	29 (8.9)	298 (91.1)	ref	0.269
	Yes	184 (36.0)	85 (46.2)	99 (53.8)	1.58 (1.1–2.29)		22 (12.0)	162 (88.0)	1.40 (0.78–2.51)	
use of Monoclonal antib.	No	408 (79.8)	126 (30.9)	282 (69.1)	ref	<0.001	40 (9.8)	368 (90.2)	ref	0.793
	Yes	103 (20.2)	74 (71.8)	29(28.2)	5.71 (3.54–9.21)		11 (10.7)	92 (89.3)	1.10 (0.54–2.23)	
Diabetes	No	430 (84.1)	162 (37.7)	268 (62.3)	ref	0.121	34 (7.9)	396 (92.1)	ref	0.001
	Yes	81 (15.9)	38 (46.9)	43 (53.1)	1.46 (0.91–2.36)		17 (21.0)	64 (79.0)	3.09 (1.63–5.86)	
Hypertension	No	295 (57.7)	96 (32.5)	199 (67.5)	ref	<0.001	16 (5.4)	279 (94.6)	ref	<0.001
	Yes	216 (42.3)	104 (48.2)	112 (51.9)	1.93 (1.34–2.76)		35 (16.2)	181 (83.8)	3.37 (1.81–6.27)	
Renal failure	No	482 (94.3)	185 (38.4)	297 (61.6)	ref	0.158	45 (9.3)	437 (90.7)	ref	0.076
	Yes	29 (5.7)	15 (51.7)	14 (48.3)	1.72 (0.81–3.65)		6 (20.7)	23 (79.3)	2.53 (0.98–6.55)	
Cancer	No	458 (89.6)	178 (38.9)	280 (61.1)	ref	0.710	41 (9.0)	417 (91.0)	ref	0.037
	Yes	53 (10.4)	22 (41.5)	31 (58.5)	1.12 (0.63–1.99)		10 (18.9)	43 (81.1)	2.37 (1.11–5.05)	
CVD	No	358 (70.1)	123 (34.4)	76 (53.1)	ref	0.027	8 (2.2)	350 (97.8)	ref	<0.001
	Yes	143 (28.0)	67 (46.9)	282 (69.1)	1.56 (1.05–2.3)		33 (23.1)	110 (76.9)	5.83 (3.16–10.77)	
COPD	No	460 (90.0)	172 (37.4)	288 (62.6)	ref	0.016	40 (8.7)	420 (91.3)	ref	0.009
	Yes	51 (10.0)	28 (54.9)	23 (45.1)	2.04 (1.14–3.65)		11 (21.6)	40 (78.4)	2.89 (1.38–6.06)	
Obesity	No	447 (87.5)	168 (37.6)	279 (62.4)	ref	0.060	37 (8.3)	410 (91.7)	ref	0.002
	Yes	64 (12.5)	32 (50.0)	32 (50.0)	1.66 (0.98–2.81)		14 (21.9)	50 (78.1)	3.10 (1.57–6.13)	
Overall	-	511 (100.0)	200 (39.1)	311 (60.9)	-	-	51 (10.0)	460 (90.0)	-	-
P/F at admission (Median–IQR) *		343 (271–406)	283 (199–344)	376 (319–438)	0.95 (0.94–0.96)	<0.001	221 (144–329)	352 (289–410)	0.95 (0.94–0.98)	<0.001

List of abbreviations: CS: Corticosteroid use; CVD: Cardiovascular disease; COPD: Chronic obstructive pulmonary disease; OR: Odds-ratio; CI: Confidence intervals; and P/F: PaO_2_/FiO_2_ ratio. * Odds ratio related to P/F at admission has to be expressed with the increment of 5 units of P/F.

**Table 2:** Number of patients in the steroid and non-steroid therapy with respect to the ARDS categories.

P/F Ratio at Admission (mmHg)	Total N (%)	CS No N (%)	CS Yes N (%)
<100	9 (1.8)	1 (11.1)	8 (88.9)
100–150	24 (4.7)	2 (8.3)	22 (91.7)
150–200	28 (5.5)	8 (28.6)	20 (71.4)
200–300	103 (20.2)	40 (38.8)	63 (61.2)
>300	347 (67.9)	260 (74.9)	87 (25.1)
Total N (%)	511 (100)	311 (60.9)	200 (39.1)

**Table 3:** Estimated margins of probability of death within 28 days with respect to P/F for exposed and unexposed cohorts. Difference column explains the steroid effect.

P/F	Steroids	Prob. of Death within 28 Days		
		Average (%) (95% CI)	Difference (%) (95% CI)	*p*-Value
60	No	36.2 (3.9–68.4)	−2.8 (−37.8; 32.2)	0.875
	Yes	33.4 (18.6–48.1)		
85	No	31.2 (3.9–58.5)	−0.3 (−30.1; 29.4)	0.982
	Yes	30.9 (18.1–43.7)		
110	No	26.7 (4.1–49.2)	1.9 (−22.9; 26.6)	0.884
	Yes	28.5 (17.6–39.5)		
135	No	22.5 (4.3–40.7)	3.7 (−16.5; 23.9)	0.716
	Yes	26.2 (17.0–35.5)		
160	No	18.8 (4.5–33.1)	5.3 (−10.8; 21.4)	0.518
	Yes	24.1 (16.3–31.9)		
185	No	15.5 (4.5–26.40)	6.6 (−6.1; 19.2)	0.309
	Yes	22.0 (15.4–28.6)		
210	No	12.6 (4.4–20.8)	7.5 (−2.4; 17.4)	0.138
	Yes	20.1 (14.4–25.70)		
235	No	10.2 (4.1–16.2)	8.1 (0.3; 15.9)	0.043
	Yes	18.3 (13.2–23.3)		
260	No	8.1 (3.6–12.6)	8.4 (1.9; 14.9)	0.011
	Yes	16.5 (11.9–21.2)		
285	No	6.4 (2.9–9.9)	8.5 (2.7; 14.3)	0.004
	Yes	14.9 (10.3–19.5)		
310	No	5.0 (2.2–7.9)	8.4 (2.9; 13.9)	0.003
	Yes	13.5 (8.8–18.2)		
335	No	3.9 (1.4–6.4)	8.2 (2.7; 13.6)	0.004
	Yes	12.1 (7.2–16.9)		
360	No	3.0 (0.8–5.3)	7.8 (2.3; 13.3)	0.006
	Yes	10.8 (5.8–15.9)		
385	No	2.3 (0.3–4.4)	7.3 (1.8; 12.9)	0.010
	Yes	9.7 (4.5–14.9)		

**Table 4:** Patients’ demographic, co-morbidities, and univariate analysis results with respect to death within 28 days or ICU. LR Test *p*-value indicates the likelihood-ratio test performed between the unadjusted model and the adjusted model with one comorbidity at time.

Patients Feature		Total N (%)	Death or ICU N (%)	Not Death or ICU N(%)	OR (95% CI)	*p*-Value	LR Test *p*-Value
Age	<50 Years	110 (21.5)	12 (10.9)	98 (89.1)	ref	0.002	0.424
	50–70 Years	198 (38.7)	31 (15.7)	167 (84.3)	1.52 (0.744–3.09)		
	>70 Years	203 (39.7)	52 (25.6)	151 (74.4)	2.81 (2.56–28.14)		
Sex	Female	181 (35.4)	33 (18.2)	148 (81.8)	ref	0.877	0.676
	Male	330 (64.6)	62 (18.8)	268 (81.2)	1.04 (0.65–1.66)		
Use of Antivirals	No	185 (36.2)	24 (13.0)	161 (87.0)	ref	0.012	0.440
	Yes	326 (63.8)	71 (21.8)	255 (78.2)	1.87 (1.13–3.09)		
Use of Aminoquinoline	No	218 (42.7)	43 (19.7)	175 (80.3)	ref	0.571	0.355
	Yes	293 (57.3)	52 (17.7)	241 (82.3)	.88 (0.56–1.36)		
Use of Azithromycin	No	327 (64.0)	52 (15.9)	275 (84.1)	ref	0.040	0.579
	Yes	184 (36.0)	43 (23.4)	141 (76.6)	1.61 (1.03–2.53)		
Use of monoclonal antib.	No	408 (79.8)	62 (15.2)	346 (84.8)	ref	<0.001	0.788
	Yes	103 (20.2)	33 (32.0)	70 (68.0)	2.63 (1.6–4.31)		
Diabetes	No	430 (84.1)	73 (17.0)	357 (83.0)	ref	0.038	0.326
	Yes	81 (15.9)	22 (27.2)	59 (72.8)	1.82 (1.05–3.16)		
Hypertension	No	295 (57.7)	41 (13.9)	254 (86.1)	ref	0.002	0.215
	Yes	216 (42.3)	54 (25.0)	162 (75.0)	2.07 (1.32–3.24)		
Renal failure	No	482 (94.3)	85 (17.6)	397 (82.4)	ref	0.036	0.202
	Yes	29 (5.7)	10 (34.5)	19 (65.5)	2.46 (1.1–5.47)		
Cancer	No	458 (89.6)	82 (17.9)	376 (82.1)	ref	0.037	0.367
	Yes	53 (10.4)	13 (24.5)	40 (75.5)	1.49 (0.76–2.91)		
CVD	No	358 (70.1)	41 (11.5)	317 (88.5)	ref	<0.001	<0.001
	Yes	143 (28.0)	44 (30.8)	99 (69.2)	2.76 (1.74–4.39)		
COPD	No	460 (90.0)	80 (17.4)	380 (82.6)	ref	0.047	0.606
	Yes	51 (10.0)	15 (29.4)	36 (70.6)	1.98 (1.03–3.79)		
Obesity	No	447 (87.5)	73 (16.3)	374 (83.7)	ref	0.001	0.013
	Yes	64 (12.5)	22 (34.4)	42 (65.6)	2.68 (1.51–4.76)		
Overall	-	511 (100.0)	95 (18.6)	416 (81.4)	-	-	
P/F at admission (Median–IQR) *		343 (271–406)	244 (148–329)	356 (296–419)	0.95 (0.94–0.96)	<0.001	-

List of abbreviations: CS: Corticosteroid use; CVD: Cardiovascular disease; COPD: Chronic obstructive pulmonary disease; OR: Odds-ratio; CI: Confidence intervals; P/F: PaO_2_/FiO_2_ ratio. * Odds ratio related to P/F at admission has to be expressed with the increment of 5 units of P/F.

**Table 5:** Estimated margins of probability of death within 28 days or ICU with respect to P/F values for exposed and unexposed cohorts. Difference column explains the steroid effect.

P/F	Steroids	Prob. of Death within 28 Days or ICU		
		Average (%) (95% CI)	Difference (%) (95% CI)	*p*-Value
60	No	59.7 (24.0–95.3)	4.5 (−34.2; 43.1)	0.821
	Yes	64.1 (48.6–79.7)		
85	No	52.1 (18.8–85.4)	9.1 (−26.9; 45.1)	0.622
	Yes	61.2 (46.7–75.6)		
110	No	44.5 (15.0–73.9)	13.7 (−18.4; 45.7)	0.403
	Yes	58.1 (44.9–71.3)		
135	No	37.1 (12.4–61.8)	17.9 (−9.3; 45.1)	0.197
	Yes	55.0 (43.0–66.9)		
160	No	30.3 (10.6–50.0)	21.6 (−0.6; 43.8)	0.056
	Yes	51.9 (41.2–62.5)		
185	No	24.2 (9.2–39.2)	24.5 (7.0; 42.0)	0.006
	Yes	48.7 (39.3–58.1)		
210	No	19.0 (8.0–29.9)	26.6 (13.0; 40.2)	<0.001
	Yes	45.6 (37.3–53.8)		
235	No	14.6 (6.8–22.5)	27.9 (17.1; 38.6)	<0.001
	Yes	42.5 (35.0–49.9)		
260	No	11.1 (5.5–16.8)	28.3 (19.4; 37.2)	<0.001
	Yes	39.4 (32.5–46.4)		
285	No	8.4 (94.2–12.5)	28.1 (20.1; 36.1)	<0.001
	Yes	36.5 (29.6–43.3)		
310	No	6.2 (2.9–9.5)	27.4 (19.6; 35.2)	<0.001
	Yes	33.6 (26.5–40.7)		
335	No	4.6 (1.9–7.3)	26.3 (18.2; 34.3)	<0.001
	Yes	30.9 (23.3–38.4)		
360	No	3.4 (1.0–5.7)	24.9 (16.4; 33.4)	<0.001
	Yes	28.3 (20.1–36.4)		
385	No	2.5 (0.5–4.4)	23.3 (14.4; 32.2)	<0.001
	Yes	25.8 (17.1–34.5)		

**Figure A1:**
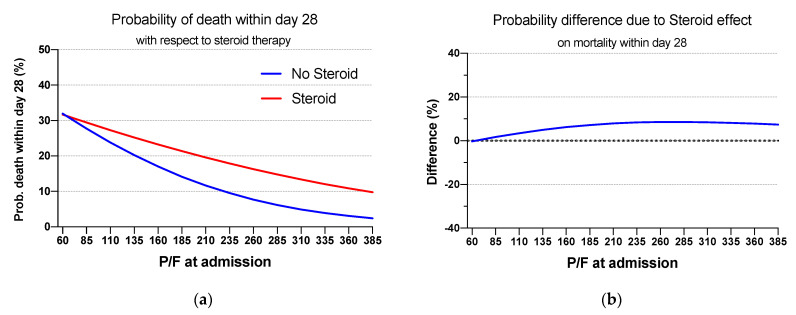
(**a**) Estimated probability of death within 28 days with respect to P/F values and steroid therapy. (**b**) Estimated difference in mortality within 28 days between exposed and unexposed cohort. Light blue shaded area shows confidence interval.

## Data Availability

The data presented in this study are available on request from the corresponding author.

## Supplementary Materials

The following are available online at https://www.mdpi.com/article/10.3390/jcm10153236/s1, Figure S1: Flow diagram of the method used for confounders selection, Figure S2: Box plot of the P/F distribution between the two cohorts: steroid treated patients and non-steroid patients, Table S1: Odds Ratios and CI for unadjusted and adjusted models involving mortality by day 28 as outcome, Figure S3: Odds ratio for mortality by day 28 adjusted model, Table S2: Odds Ratios and CI for unadjusted and adjusted model involving mortality by day 28 or Intensive Care Unit admission as combined outcome, Figure S4: Odds ratio for mortality by day 28 or Intensive Care Unit admission adjusted model.

## Appendix A

In this section the results of a sensitivity analysis involving age as a continuous variable are presented.

The analysis is the same as that shown in Section 3.3. We used 28-day all-cause mortality as the outcome and every other predictor involved in Section 3.3 except the age variable, which in this case has been involved as a continuous variable.

Overall, the steroid-treated cohort shows a median (IQR) age of 69 (58–78) years, while the untreated cohort shows a median (IQR) age of 61 (50–75). Even in this case, the average marginal predictions have been calculated in order to return the heterogeneous effect of steroid therapy on the probability of the outcome, as shown in Table A1.

**Table A1:** Estimated margins of probability of death within 28 days with respect to P/F for exposed and unexposed cohorts. Difference column explains the steroid effect.

P/F	Steroids	Prob. of Death within 28 Days		
		Average (%) (95% CI)	Difference (%) (95% CI)	*p*-Value
60	No	31.9 (2.7–61.2)	−0.3 (−32.2–31.6)	0.986
	Yes	31.7 (18.2–45.2)		
85	No	27.7 (2.9–52.5)	1.7 (−25.5–28.9)	0.903
	Yes	29.4 (17.7–41.2)		
110	No	23.8 (3.3–44.4)	3.5 (−19.2–26.1)	0.765
	Yes	27.3 (17.2–37.3)		
135	No	20.2 (3.6–36.9)	5.0 (−13.6–23.5)	0.600
	Yes	25.2 (16.7–33.8)		
160	No	17. (3.9–30.2)	6.2 (−8.7–21.1)	0.413
	Yes	23.2 (16–30.4)		
185	No	14.2 (4–24.3)	7.2 (−4.6–18.9)	0.231
	Yes	21.3 (15.2–27.5)		
210	No	11.7 (4–19.3)	7.9 (−1.4–17.1)	0.095
	Yes	19.5 (14.3–24.8)		
235	No	9.5 (3.8–15.3)	8.3 (0.9–15.7)	0.027
	Yes	17.8 (13.1–22.6)		
260	No	7.7 (3.3–12.)	8.6 (2.4–14.8)	0.007
	Yes	16.3 (11.8–20.7)		
285	No	6.2 (2.8–9.6)	8.6 (3.0–14.2)	0.003
	Yes	14.8 (10.3–19.2)		
310	No	4.9 (2.1–7.7)	8.5 (3.1–13.8)	0.002
	Yes	13.4 (8.8–17.9)		
335	No	3.9 (1.4–6.3)	8.2 (2.8–13.6)	0.003
	Yes	12.1 (7.3–16.8)		
360	No	3.1 (0.8–5.3)	7.8 (2.4–13.3)	0.005
	Yes	10.9 (5.9–15.8)		
385	No	2.4 (0.4–4.4)	7.4 (1.8–12.9)	0.009
	Yes	9.8 (4.6–14.9)		

As we can see from Table A1 and Figure A1, the estimated difference between treated and untreated patients is the same as the one seen in the multivariable logistic regression model adjusted with age as a categorical variable.
