# Supplementary material for: Benefits of Steroid Therapy in COVID-19 Patients with Different PaO2/FiO2 Ratio at Admission
Source: J Clin Med. 2021 Jul 22;10(15):3236. doi: 10.3390/jcm10153236 (PMC8347049; doi:10.3390/jcm10153236)
Supplement: Supplementary file 1 [file jcm-10-03236-s001.zip › jcm-1289139-supplementary.pdf]

Journal of
Clinical Medicine

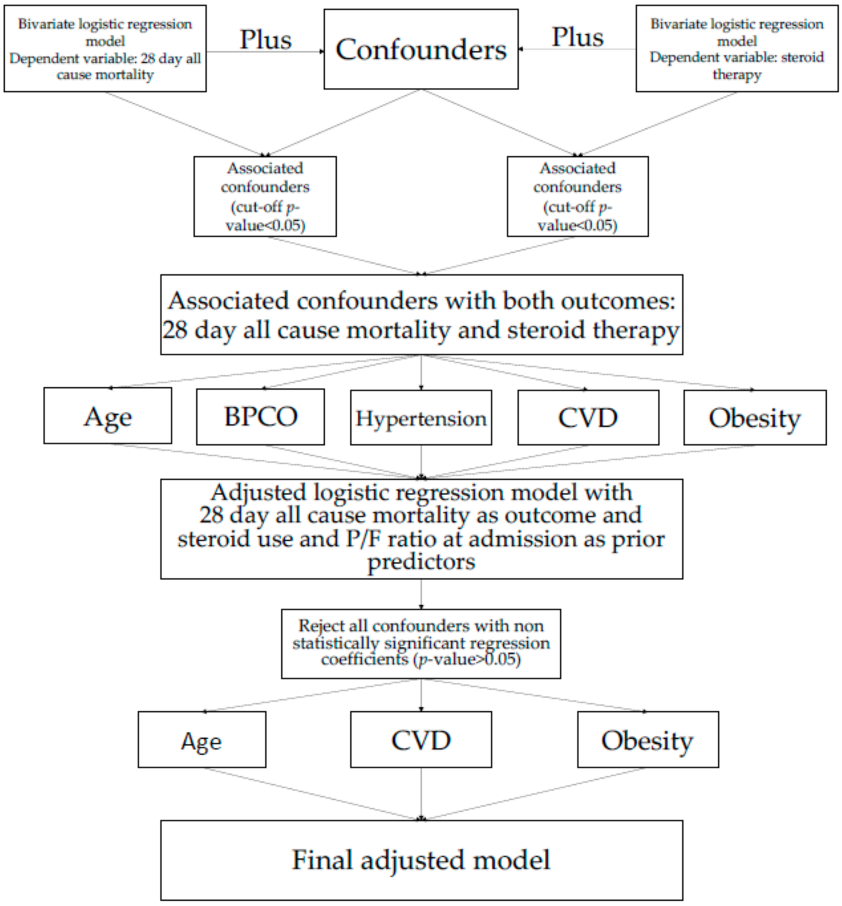

Figure S1. Flow diagram of the method used for confounders selection.

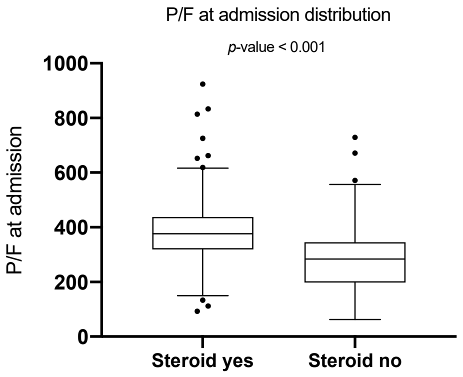

Figure S2. Box plot of the P/F distribution between the two cohorts: steroid treated patients and non steroid patients.

Table S1. Odds Ratios and CI for unadjusted and adjusted model involving death within 28 days as outcome.

Patients feature		Total N (%)	Deceased N(%)	Unadjusted model		Adjusted model	
				OR (95% CI)	p-value	OR (95% CI)	p-value
CVD	No	358 (70.1)	8 (2.2)			ref	
	Yes	143 (28.0)	33 (23.1)			3.97 (1.89 - 8.32)	<0.001
Obesity	No	447 (87.5)	37 (8.3)			ref	
	Yes	64 (12.5)	14 (21.9)			3.60 (1.58 - 8.23)	0.002
Age	<50 Years	110 (21.5)	3 (2.7)			ref	-
	50-70 Years	198 (38.7)	9 (4.6)			0.68 (0.16 - 2.83)	0.592
	>70 Years	203 (39.7)	39 (19.2)			2.18 (0.58 - 8.24)	0.250
use of Steroid	No	311 (60.9)	12 (3.9)	ref		ref	
	Yes	200 (39.1)	39 (19.5)	0.38 (0.04 - 3.34)	0.383	0.62 (0.06 - 6.69)	0.691
Overall	-	511 (100.0)	51 (10.0)				
P/F at admission (Median - IQR)		343 (271 - 406)	352 (288.5 - 410)	0.99 (0.98 - 0.99)	<0.001	0.99 (0.98 - 1.00)	0.002
P/F at admission * Steroid use		-	-	1.01 (1.00 - 1.01)	0.061	1.01 (1.00 - 1.01)	0.182

[1] List of abbreviations: CVD: Cardiovascular disease; COPD: Chronic obstructive pulmonary disease; OR: Odds-ratio; CI: Confidence intervals; P/F: PaO_2/FiO_2 ratio.

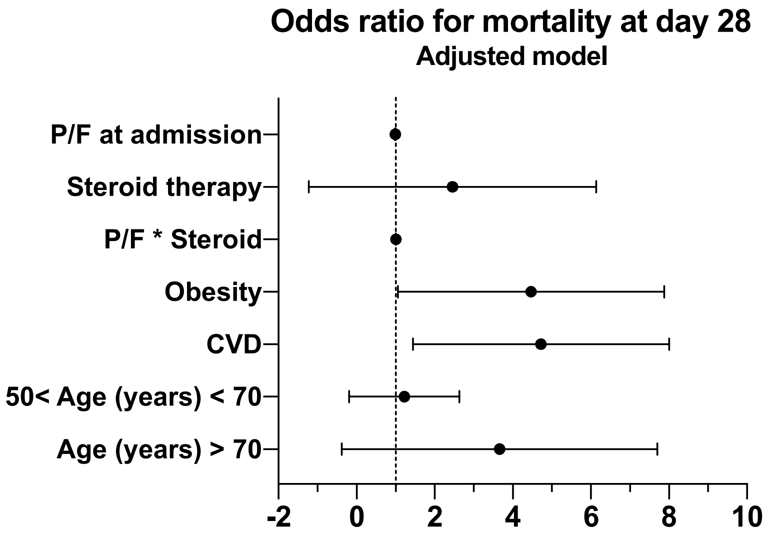

Figure S3. Odds ratio related to the adjusted model involving mortality at day 28 as outcome.**Table S2.** Odds Ratios and CI for unadjusted and adjusted model involving death within 28 days or Intensive Care Unit admission as combined outcome.

**Patients feature**		**Total N (%)**	**Death or ICU N (%)**	**Unadjusted model OR (95% CI)**	***p*-value**	**Adjusted model OR (95% CI)**	***p*-value**
**CVD**	No	358 (70.1)	41 (11.5)			ref	0.002
	Yes	143 (28.0)	44 (30.8)			2.75 (1.46 - 5.18)	
**Obesity**	No	447 (87.5)	73 (16.3)			ref	0.009
	Yes	64 (12.5)	22 (34.4)			2.61 (1.27 - 5.34)	
**Age**	<50 Years	110 (21.5)	12 (10.9)			ref	-
	50-70 Years	198 (38.7)	31 (15.7)			0.76 (0.33 - 1.76)	0.520
	>70 Years	203 (39.7)	52 (25.6)			0.77 (0.32 - 1.84)	0.562
**Use of Steroid**	No	311 (60.9)	15 (4.8)	ref	0.695	ref	0.806
	Yes	200 (39.1)	80 (40.0)	0.67 (0.09 - 5.04)		0.77 (0.09 - 6.36)	
**Overall**	-	511 (100.0)	95 (18.6)				
**P/F at admission (Median - IQR)**		343 (271 - 406)	356 (296 - 419)	0.99 (0.98 - 0.99)	<0.001	0.99 (0.98 - 0.99)	<0.001
**P/F at admission * Steroid use**		-	-	1.01 (1.00 - 1.02)	0.02	1.01 (1.00 - 1.01)	0.033

[1] List of abbreviations: CVD: Cardiovascular disease; COPD: Chronic obstructive pulmonary disease; OR: Odds-ratio; CI: Confidence intervals; P/F: PaO_2/FiO_2 ratio.

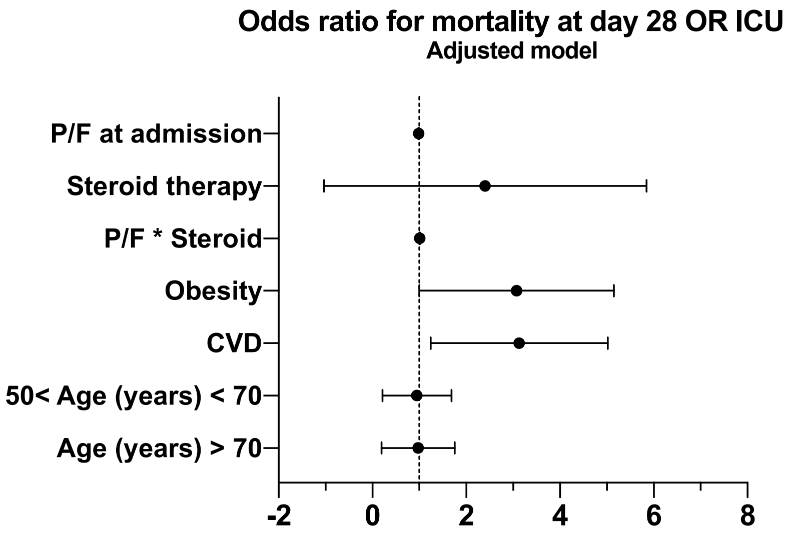

Figure S4. Odds ratio related to the adjusted model involving mortality at day 28 or Intensive Care Unit admission as combined outcome
